# The effect of overnight culture after thawing of D3 cleavage-stage embryos on clinical pregnancy outcomes: focus on embryo development to day 4

**DOI:** 10.3389/fendo.2025.1530112

**Published:** 2025-05-01

**Authors:** Liuping Lan, Xiang Li, Bowen Luo, Weiwu Liu, Lingling Zhu, Juguang Zhang, Jian Wen, Keng Feng, Derong Li, Feifei Lei, Guosheng Deng, Yudi Luo, Zengyu Yang

**Affiliations:** Reproductive Medicine Center, Maternal and Child Health Care Hospital of Yulin, Yulin, Guangxi, China

**Keywords:** D3 cleavage-stage embryo, frozen embryo transfer (FET), overnight culture, embryo development, clinical pregnancy outcomes

## Abstract

**Objective:**

This study aims to investigate the impact of day-3 (D3) cleavage-stage embryo thawing with immediate transfer versus thawing and overnight culture before transfer on clinical outcomes. It also examines the relationship between cleavage-stage embryo developmental speed after overnight culture and clinical pregnancy outcomes, as well as factors influencing clinical pregnancy in frozen embryo transfer (FET).

**Methods:**

A retrospective analysis was conducted on 1,040 patients who underwent D3 cleavage-stage frozen embryo transfer at Yulin City Maternal and Child Health Hospital between July 2022 and December 2023. Patients were divided into two groups based on embryo culture time after thawing: control (same-day transfer, 2-3 hours) and experimental (overnight culture, 18-20 hours). Clinical pregnancy rates, embryo implantation rates, early miscarriage rates, and multiple pregnancy rates were compared between groups. The experimental group was further subdivided based on the number of cleavage blastomeres increased after culture: A1 (≥4 blastomeres), A2 (1-3 blastomeres), and A3 (no increase). A binary logistic regression analysis identified independent factors affecting clinical pregnancy outcomes in FET.

**Results:**

No significant differences were found between the control and experimental groups in clinical pregnancy rate (37.2% vs. 40.2%), embryo implantation rate (24.9% vs. 26.4%), early miscarriage rate (13.1% vs. 18.8%), or multiple pregnancy rate (9.2% vs. 10.2%) (*P* > 0.05). In the experimental group, clinical pregnancy rates for A1, A2, and A3 subgroups were 44.2%, 29.8%, and 25.5%, respectively. Early miscarriage rates were 18.6%, 10.7%, and 38.5%, showing statistically significant differences (*P* < 0.05). Female age, endometrial thickness, embryo morphology, and the number of cleavage blastomeres were identified as independent factors influencing clinical pregnancy rate.

**Conclusion:**

This study indicates that D3 embryos with an increase in the number of blastomeres to more than four or entering the compaction stage after overnight culture have better pregnancy outcomes. Female age and endometrial thickness are important factors influencing clinical pregnancy rates. Optimizing culture conditions and ensuring optimal endometrial thickness may help improve the success rate of frozen-thawed embryo transfer.

## 1 Introduction

With advancements in assisted reproductive technology (ART), vitrification techniques have become increasingly refined, and the application of cryopreservation in *in vitro* fertilization (IVF) labs has improved survival rates of oocytes, cleavage-stage embryos, and blastocysts after thawing. This has led to the widespread adoption of frozen-thawed embryo transfer (FET) (1). Data indicates that over the past decade, the number of FET cycles has increased significantly, accounting for more than 30%-40% of all transfer cycles in many regions worldwide (2).

FET not only allows patients to choose an optimal time for embryo transfer, helps prevent ovarian hyperstimulation syndrome, and reduces embryo waste, but it also plays a key role in decreasing multiple pregnancy rates and increasing cumulative live birth rates (3, 4). Furthermore, compared to fresh embryo transfer, FET has been shown to improve implantation, clinical pregnancy, and ongoing pregnancy rates in ART cycles (5), while posing lower risks of placenta previa, placental abruption, low birth weight, very low birth weight, preterm birth, small-for-gestational age, and perinatal mortality (6).

Factors influencing clinical outcomes of FET include maternal age, BMI, embryo quality, endometrial thickness on the day of transfer, number of embryos transferred, and post-thaw recovery of embryos (7). However, optimizing post-thaw outcomes for cleavage-stage embryos remains controversial, with no consensus yet reached. In clinical practice, the *in vitro* culture duration after D3 cleavage-stage embryo thawing varies, and its impact on pregnancy outcomes is still debated (8).

Our study is novel in that it provides a detailed analysis of the clinical outcomes of Day 3 cleavage-stage embryos subjected to overnight culture (to Day 4) after thawing, compared to embryos transferred shortly after thawing. Specifically, we assessed the developmental potential of embryos during the overnight culture and categorized them based on the extent of blastomere division into subgroups (A1, A2, and A3). This level of granularity in embryo classification, combined with a large sample size, allows for a nuanced understanding of how post-thaw developmental progression impacts clinical outcomes.

Therefore, this study retrospectively analyzed 1040 FET cycles at our center, all involving D3 cleavage-stage embryo transfers after thawing, to investigate the relationship between *in vitro* culture duration after D3 embryo thawing and clinical pregnancy outcomes, as well as the impact of different embryo developmental rates following overnight culture. This analysis also considers additional factors influencing clinical pregnancy outcomes in FET.

## 2 Materials and methods

### 2.1 Study subjects and grouping

A retrospective analysis was conducted on medical records of patients who underwent D3 cleavage-stage frozen-thawed embryo transfer (FET) at our center from July 2022 to December 2023, encompassing a total of 1040 cycles. Ethical approval for this study was obtained from the ethics committee, and the approval number was YLSFYLLKY2025-02-05-1.Inclusion criteria were female age <50 years and male age <60 years, with infertility factors such as female pelvic/tubal or ovulatory disorders, and male oligoasthenoteratozoospermia. IVF or ICSI was performed. Exclusion criteria included FET cycles with sequential cleavage-stage and blastocyst transfer, egg recipients, and patients who underwent oocyte vitrification followed by embryo transfer.

The cycles were grouped based on the timing of thawing. The control group (470 cycles) involved thawing in the morning with embryo transfer approximately 2 hours later. The experimental group (570 cycles) underwent thawing in the afternoon of the previous day, followed by an 18–20 hour overnight culture prior to transfer.

### 2.2 Methods

#### 2.2.1 Embryo cryopreservation and thawing

The KITAZATO cryopreservation and thawing kits (Japan) and Vitrolife sequential culture media were used.

##### 2.2.1.1 Embryo vitrification

At room temperature, embryos were transferred from G1 medium to the equilibration solution (ES) and equilibrated for 10 minutes until approximately 90% of blastomeres recovered. Embryos were then transferred to vitrification solution (VS) and, within 60 seconds, loaded onto cryo-straws and preserved in liquid nitrogen.

##### 2.2.1.2 Embryo thawing

Cryo-straws containing embryos were transferred directly from liquid nitrogen to WS1 thawing solution prewarmed to 37°C, shaken gently to ensure release of the embryo into the solution, and kept in the solution for 1 minute. The embryos were subsequently moved through WS2, WS3, and WS4 solutions sequentially (3 min, 5 min, 5 min), with WS2 and WS3 at room temperature and WS1 and WS4 at 37°C. Embryos were then cultured overnight in microdrops of G2 medium within a tri-gas incubator (6% CO_2_, 5% O_2_, balanced with N_2_) to await transfer.

#### 2.2.2 Endometrial preparation

Endometrial preparation includes the natural cycle, ovulation induction cycle, and rtificial cycle.

##### 2.2.2.1 Natural cycle

Starting from day 10 of menstruation, monitor follicle development with ultrasound until the dominant follicle diameter is ≥16mm. At this point, begin monitoring blood levels of estradiol, progesterone, and luteinizing hormone. On the day of ovulation, administer oral dydrogesterone 10mg tid (Duphaston, 10mg/tablet, Abbott Biologicals B.V.) or progesterone capsules 200mg bid (Yimaixin, 50mg/pill, Zhejiang Xianju) for luteal support (D0). Embryo transfer on day 3.

##### 2.2.2.2 Ovulation induction cycle

Start oral administration of letrozole (2.5 mg per tablet, Jiangsu Hengrui) at 2.5 mg to 5 mg once daily for 5 days starting from day 2 to day 5 of menstruation. Begin monitoring ovulation 5 days later. If two monitored follicles are ≤10 mm, add HMG 75-150 IU until the leading follicle diameter is ≥16 mm. Then start monitoring blood E2, P, and LH levels. Once natural ovulation occurs or if the follicle does not rupture but P exceeds 1 ng/ml, administer orally: 1. Dydrogesterone 10 mg three times daily (Duphaston, 10 mg per tablet, Abbott Biologicals B.V., Netherlands) 2. Progesterone capsules 200 mg twice daily (Yimaixin, 50 mg per tablet, Zhejiang Xianju) for luteal support (Day 0). Transfer on Day 5.

##### 2.2.2.3 Artificial cycle

Start oral administration of estradiol valerate (Juvacon, 1 mg/tablet, Bayer France, DELPHARM Lille S.A.S.) or Femilon red tablets (each red tablet equivalent to 1 mg of estradiol valerate, Abbott Biologicals B.V., Netherlands) from the second to third day of menstruation. Once the endometrial thickness reaches ≥8 mm, administer progesterone injection 40 mg daily (20 mg/vial, Zhejiang Xianju) for luteal support (D0). Embryo transfer is performed on the D3.

#### 2.2.3 Embryo transfer

D3 cleavage-stage embryos were considered viable for transfer if at least half of the blastomeres survived; embryos with more than half of the blastomeres degenerated were not transferred.

##### 2.2.3.1 Control group

Embryos were thawed in the morning and transferred into the G2 medium culture dish for 2 hours before transfer.

##### 2.2.3.2 Experimental group

Embryos were thawed the previous afternoon and cultured in G2 medium for 18–20 hours overnight, and the development status was observed the following morning before transfer.

### 2.3 Outcome measures

#### 2.3.1 D3 embryo assessment

Grade I: Derived from normal fertilization, 8 cells of uniform size with ≤5% fragmentation; Day 2 (D2) embryo quality is Grade I or II, with 4 cells. Grade II: Derived from normal fertilization, 7–9 cells, with a cell count increase of more than 2 cells from D2, uniform or slightly uneven cell size, and ≤10% fragmentation; D2 embryo quality is Grade I or II. Grade III: ≥5 cells with a cell count increase of at least 2 cells from D2, moderate cell uniformity (≤++), and fragmentation between 5% and 50%. Grade IV: ≤4 cells, or severe cell size unevenness, with ≥50% fragmentation. Grades I and II are collectively classified as high-quality embryos; Grade III is classified as non-high-quality, and Grades I, II, and III are considered usable embryos.

#### 2.3.2 Thawing and culture protocol

The cycles were grouped based on the timing of embryo thawing. The control group (470 cycles) involved thawing in the morning with embryo transfer approximately 2 hours later. The experimental group (570 cycles) underwent thawing in the afternoon of the previous day, followed by an 18–20 hour overnight culture prior to transfer. In the experimental group, embryos were further divided into three subgroups based on the number of blastomeres observed the next morning:

Group A1: Embryos with more than 4 cells were assessed for further cleavage and developmental potential. For embryos entering the compaction stage, individual cell counts were not distinguishable due to the tightly packed arrangement of cells. In this study, compacted embryos were evaluated as part of the cleavage-stage developmental continuum, without being categorized separately. The primary focus was on assessing overall morphological development and clinical outcomes rather than subdividing specific morphological stages.
Group A2: Embryos with 1–3 new blastomeres.
Group A3: Embryos with no increase in blastomeres.

#### 2.3.3 Other metrics and pregnancy determination

Post-Thaw Embryo Survival Rate: Number of surviving thawed embryos / total number of thawed embryos. Post-Thaw Intact Embryo Rate: Number of intact thawed embryos / total number of thawed embryos. Fourteen days post-transfer, blood hCG is measured, and clinical pregnancy is confirmed via ultrasound at 28 days if a gestational sac is observed. Clinical Pregnancy Rate: Number of clinical pregnancy cycles / total transfer cycles × 100%. Embryo Implantation Rate: Number of implanted embryos / total number of transferred embryos × 100%. Early Miscarriage Rate: Natural miscarriages within 12 weeks of gestation / number of clinical pregnancy cycles × 100%. Multiple Pregnancy Rate: Number of multiple pregnancies / number of clinical pregnancies × 100%.

### 2.4 Statistical methods

Data were analyzed using SPSS 25.0 software. Continuous variables were presented as mean ± standard deviation (x ± s) and analyzed with the t-test. Categorical variables were expressed as percentages (%) and analyzed with the χ² test. A p-value <0.05 was considered statistically significant. Binary logistic regression was conducted to assess the correlation between the increase in blastomere number and clinical pregnancy rate, with odds ratios (OR) and 95% confidence intervals (CI) calculated.

## 3 Results

### 3.1 Comparison of basic characteristics between the control and experimental groups

There were no statistically significant differences between the control and experimental groups in terms of the average age of the female participants, duration of infertility, body mass index (BMI), endometrial thickness on the day of embryo transfer, average number of embryos transferred, embryo survival rate and integrity after thawing, endometrial preparation protocol, and embryo morphology (*P* > 0.05), (**Table 1**).

**Table 1:** Comparison of basic characteristics between the control and experimental groups.

	Control group	Experimental group	t/χ^2^	*P*
Number of cycles	470	570		
Average age of female partner (years)	36.80 ± 5.08	37.25 ± 5.19	-1.413	0.856
Infertility duration (years)	4.89 ± 3.46	4.83 ± 3.69	0.542	0.430
body mass index (kg/m²)	22.56 ± 3.21	22.72 ± 3.22	-0.802	0.515
Inner membrane thickness (mm)	9.80 ± 1.72	9.97 ± 1.83	-1.555	0.155
Average number of embryos transferred	1.87 ± 0.34	1.87 ± 0.32	-0.626	0.211
Survival rate of revived embryos (%)	98.88	98.69	0.138	0.710
Recovery embryo integrity rate (%)	97.08	96.77	0.151	0.697
Infertility type			2.031	0.154
Primary infertility	139	146		
Secondary infertility	331	424		
Endometrial preparation plan			7.550	0.056
Natural cycle	89	100		
Ovulation induction cycle	70	82		
Hormone replacement cycle (GnRH-α+HRT)	91	151		
Hormone replacement cycle (HRT)	220	237		
Transplanted embryo morphology			4.148	0.126
Excellent embryo	275	333		
Excellent embryo +non excellent embryo	114	161		
non excellent embryo	81	76		

### 3.2 Comparison of cinical outcomes between the control and experimental groups

In the experimental group (570 cycles), the clinical pregnancy rate was 40.20%, the embryo implantation rate was 26.40%, the early miscarriage rate was 18.80%, and the multiple pregnancy rate was 10.18%. In the control group (470 cycles), the clinical pregnancy rate was 37.20%, the embryo implantation rate was 24.90%, the early miscarriage rate was 13.10%, and the multiple pregnancy rate was 9.15%. The clinical pregnancy rate, implantation rate, early miscarriage rate, and multiple pregnancy rate were slightly higher in the experimental group compared to the control group, but the differences were not statistically significant (*P* > 0.05) (**Table 2**).

**Table 2:** Comparison of clinical outcomes between the control and experimental groups.

	Clinical pregnancy rate (%)	Implantation rate (%)	Early miscarriage rate (%)	Multiple birth rate (%)
control group	37.20 (175/470)	24.90 (218/877)	13.10 (23/175)	9.15 (43/470)
Experimental group	40.20 (229/570)	26.40 (283/1071)	18.80 (43/229)	10.18 (58/570)
χ^2^	0.938	0.619	2.304	0.310
*p*	0.333	0.431	0.129	0.578

### 3.3 Relationship between embryo development speed and clinical pregnancy outcomes in the pre-embolization thaw group

The results showed that there were no statistically significant differences in basic characteristics such as female age, duration of infertility, body mass index, endometrial thickness on the day of embryo transfer, and infertility type between the A1, A2, and A3 groups (P > 0.05). However, there were statistically significant differences between the groups in terms of average number of embryos transferred (*P* = 0.048), endometrial preparation protocol (P = 0.019), and embryo morphology (*P* = 0.00), while the difference in multiple pregnancy rates was not statistically significant (*P* > 0.05).

Regarding the clinical pregnancy rate, the A1, A2, and A3 groups had rates of 44.2%, 29.8%, and 25.5%, respectively, with significant differences between the three groups (*P* = 0.003). Further pairwise comparisons showed that the differences in clinical pregnancy rates between the A1 and A2 groups (χ2 = 6.613, p = 0.01) and between the A1 and A3 groups (χ2 = 6.559, *P* = 0.01) were statistically significant, but the difference between the A2 and A3 groups was not significant (χ2 = 0.301, *P* = 0.583).

For embryo implantation rates, the A1, A2, and A3 groups had rates of 29.3%, 18.3%, and 17.9%, respectively, with significant differences between the three groups (*P* = 0.002). Further pairwise comparisons revealed that the differences in implantation rates between the A1 and A2 groups (χ2 = 8.859, *P* = 0.003) and between the A1 and A3 groups (χ2 = 5.442, *P* = 0.02) were statistically significant, while the difference between the A2 and A3 groups was not significant (χ2 = 0.008, *P* = 0.929).

As for early miscarriage rates, the A1, A2, and A3 groups had rates of 18.6%, 10.7%, and 38.5%, respectively, with no statistically significant differences between the three groups (*P* = 0.129). Pairwise comparisons showed that there were no significant differences in early miscarriage rates between the A1 and A2 groups, and between the A1 and A3 groups (*P* > 0.05), but the difference between the A2 and A3 groups was statistically significant (χ2 = 4.352, *P*= 0.037) (**Table 3**).

**Table 3:** Relationship between embryo development speed and clinical pregnancy outcomes in the pre-embolization thaw group.

	A1 group (≥4 cells)	A2 group (1-3 cells)	A3 group (No increase)	F/χ^2^	*p*
Number of cycles	425	94	51		
Average age of female partner (years)	37.09 ± 5.19	38.07 ± 4.76	37.12 ± 5.81	1.388	0.251
Infertility duration (years)	4.83 ± 3.70	4.75 ± 3.63	4.35 ± 3.36	1.148	0.318
body mass index (kg/m²)	22.72 ± 3.31	22.85 ± 3.07	22.54 ± 2.78	0.456	0.634
Inner membrane thickness (mm)	10.03 ± 1.83	9.89 ± 1.83	9.55 ± 1.82	0.522	0.593
Average number of embryos transferred	1.87 ± 0.33	1.91 ± 0.28	1.86 ± 0.35	3.047	0.048
Infertility type				1.417	0.492
Primary infertility	109	21	16		
Secondary infertility	316	73	35		
Endometrial preparation plan				15.191	0.019
Natural cycle	67	24	9		
Ovulation induction cycle	65	12	5		
Hormone replacement cycle (GnRH-α+HRT)	126	17	8		
Hormone replacement cycle (HRT)	167	41	29		
Transplanted embryo morphology				36.149	0.00
Excellent embryo	273	49	11		
Excellent embryo +non excellent embryo	103	30	28		
non excellent embryo	49	15	12		
Multiple birth rate (%)	11.53	5.32	7.84	3.581	0.167
Clinical pregnancy rate (%)	44.2 (188/425)	29.8 (28/94)	25.5 (13/51)	11.711	0.003
Implantation rate (%)	29.3 (233/796)	18.3 (33/180)	17.9 (17/95)	12.935	0.002
Early miscarriage (%)	18.6 (35/188)	10.7 (3/28)	38.5 (5/13)	4.099	0.129

### 3.4 Multivariate logistic regression analysis of factors influencing clinical pregnancy in the experimental group

In the multivariate logistic regression analysis, female age and endometrial thickness were found to have a significant impact on clinical pregnancy. Increasing female age significantly decreased the likelihood of pregnancy (OR = 0.892, *P* = 0.000), while higher endometrial thickness significantly increased the pregnancy rate (OR = 1.174, *P* = 0.002). Other factors, such as duration of infertility, body mass index (BMI), number of embryos transferred, endometrial preparation protocol, and embryo morphology (good or poor quality), did not show a significant impact on clinical pregnancy (**Table 4**).

**Table 4:** Multivariate logistic regression analysis of factors influencing clinical pregnancy in the experimental group.

	Non pregnancy group	Pregnancy group	OR (95%CI)	*P*
Number of cycles	341	229		
Average age of female partner (years)	38.466 ± 5.188	35.450 ± 4.639	0.892 (0.859-0.927)	0.000
Infertility duration (years)	4.829 ± 3.770	4.688 ± 3.478	1.011 (0.961-1.064)	0.676
Body mass index (kg/m²)	22.951 ± 3.046	22.380 ± 3.447	0.950 (0.898-1.005)	0.076
Average age of female partner (years)	9.770 ± 1.815	10.259 ± 1.822	1.174 (1.060-1.299)	0.002
Average number of embryos transferred	1.874 ± 0.332	1.877 ± 0.318	0.984 (0.558-1.737)	0.957
Endometrial preparation plan n (%)				
Natural cycle	60 (17.5)	40 (17.4)	–	–
Ovulation induction cycle	41 (12.0)	41 (17.9)	1.235 (0.651-2.342)	0.519
Hormone replacement cycle (GnRH-α+HRT)	95 (27.8)	56 (24.4)	0.906 (0.518-1.586)	0.730
Hormone replacement cycle (HRT)	145 (42.5)	92 (40.1)	1.126 (0.671-1.891)	0.653
Transplanted embryo morphology n (%)				
non excellent embryo	56 (16.4)	20 (8.7)	–	–
Excellent embryo +non excellent embryo	100 (29.3)	61 (26.6)	1.589 (0.831-3.038)	0.161
Excellent embryo	185 (54.2)	148 (64.6)	1.889 (1.046-3.404)	0.034
Number of blastomeres growing n (%)				
No growth	38 (11.1)	13 (5.6)	–	–
1-3 cells	66 (19.3)	28 (12.2)	1.316 (0.571-3.031)	0.519
≥4 cells	237 (69.5)	188 (82.0)	2.208 (1.064-4.581)	0.033

## 4 Discussion

Optimizing embryo freezing, thawing, and transfer protocols is crucial for improving clinical outcomes in frozen embryo transfer (FET). The *in vitro* culture t duration of D3 cleavage-stage embryos post-thawing influences cryoprotectant removal, developmental potential, and activation. However, the optimal parameters for this process remain undetermined. Some studies suggest that pre-thawing and overnight culture of D3 cleavage-stage embryos do not significantly increase implantation or clinical pregnancy rates (9, 10). Conversely, other studies propose that restoring embryo developmental potential requires mitotic recovery, which may necessitate a longer duration, and that overnight culture could improve clinical pregnancy outcomes (11, 12). Our findings indicate that, compared to the same-day thawing group, overnight culture of D3 embryos post-thawing did not significantly enhance clinical pregnancy or implantation rates, nor did it affect early miscarriage or multiple pregnancy rates.

This outcome may be attributed to the restoration of amino acid metabolism to pre-freezing levels within the first hour after embryo thawing. In FET cycles, embryos typically recover within 2 to 3 hours post-thawing and subsequently develop normally. Thus, prolonging *in vitro* culture duration did not significantly impact embryo quality (13, 14). Moreover, prolonged *in vitro* culture may negatively affect embryos. Research indicates that an imbalance between reactive oxygen species (ROS) and antioxidants, resulting in oxidative stress (OS), is a critical factor influencing assisted reproductive technology (ART) outcomes (15). External factors, including oxygen levels, CO2 concentration, and temperature, may trigger excessive ROS production, compromising embryonic cell functions and ultimately impairing embryo development and quality. Prolonged *in vitro* culture time may also impair the embryo’s DNA repair mechanisms. While the *in vitro* environment partially replicates conditions within the fallopian tube and uterus, the *in vivo* setting remains more favorable for embryo development. Thus, timely embryo-maternal interaction may better support embryo development and enhance endometrial receptivity.

Although this study did not demonstrate that overnight culture improved FET clinical pregnancy outcomes for D3 embryos, an extended culture period of 18-20 hours led to more than 90% of embryos exhibiting varying degrees of cleavage ball proliferation. In the experimental group, embryos displaying an increase of ≥4 cleavage balls had higher clinical pregnancy and implantation rates, along with a significantly lower early miscarriage rate compared to embryos without cleavage ball proliferation. This finding suggests that embryos with restored cleavage ball division may possess greater developmental potential and higher pregnancy success rates. Previous studies suggest that embryos with at least two cleavage balls restored after overnight culture are more likely to achieve favorable clinical pregnancy outcomes (13). Following D3 embryo thawing, overnight culture Following survival rate, mitotic recovery, cleavage ball number, symmetry, and fragmentation, aiding in the assessment of developmental potential and prediction of FET outcomes (16). An increase in cleavage ball count indicates greater embryonic developmental potential, making embryo selection based on this criterion beneficial for enhancing clinical pregnancy and implantation rates while reducing early miscarriage rates (17). Embryos failing to exhibit cleavage ball proliferation post-thawing often present chromosomal abnormalities, with only a 20% likelihood of normal chromosomal composition. The transfer of such embryos may result in implantation failure or early miscarriage (18).

A study on the transfer of frozen embryos thawed on Day 2 and Day 3 showed that for embryos frozen on Day 2, there was no statistically significant difference in implantation rate, clinical pregnancy rate, or live birth rate between immediate transfer after thawing and transfer after overnight culture to Day 3 (19). Our study similarly found that for cleavage-stage embryos frozen on Day 3, whether they were transferred immediately after thawing or after overnight culture, there was no statistically significant difference in clinical pregnancy outcomes. This suggests that extending the *in vitro* culture time in frozen-thawed embryo transfer (FET) cycles may not significantly improve pregnancy outcomes, and the optimal timing of embryo transfer should be determined based on individualized clinical factors.

Furthermore, in FET cycles, blastocyst transfer is generally associated with higher clinical pregnancy and live birth rates compared to cleavage-stage embryo transfer (20). However, blastocyst transfer has also been linked to an increased incidence of pregnancy-induced hypertension, placental abnormalities, perinatal mortality, preterm birth, and large-for-gestational-age infants (21). A potential mechanism for these adverse effects is that prolonged *in vitro* culture alters the epigenetic programming of the embryo due to changes in culture medium composition and oxygen tension, potentially impacting neonatal outcomes. Additionally, during assisted reproductive technology (ART) procedures, suboptimal culture conditions may prevent some genetically normal embryos from developing into blastocysts. Consequently, extended culture may lead to the loss of potentially viable embryos that could have been cryopreserved and used clinically.

This study offers a novel perspective by analyzing the clinical outcomes of Day 3 cleavage-stage embryos cultured overnight (to Day 4) after thawing. While previous studies have shown that Day 4 morula-stage embryos have higher implantation potential than embryos transferred immediately after thawing, our findings add depth by identifying subgroups based on blastomere division during overnight culture. Embryos with ≥4 divisions showed better clinical pregnancy rates and lower miscarriage rates, emphasizing the value of morphological assessment. Although some studies report no significant differences between short-term and long-term culture (8, 10), our results align with research indicating that extended culture enhances embryo selection by identifying those with greater developmental potential (16).

In this study, the correlation between factors such as female age, duration of infertility, and endometrial thickness with clinical pregnancy rate was analyzed. Previous studies have pointed out that factors such as female age, endometrial thickness on the day of transfer, body mass index (BMI), and the number of embryos transferred can all influence the outcomes of FET (22). The results of this study suggest that female age is negatively correlated with clinical pregnancy rate. As age increases, the probability of embryo-endometrial synchrony decreases, the likelihood of chromosomal abnormalities in the embryos increases, and the clinical pregnancy rate decreases. Endometrial thickness is a commonly used indicator for predicting endometrial receptivity. Studies have shown that in FET cycles, when the endometrial thickness is less than 7mm, the clinical pregnancy rate and live birth rate significantly decrease (23). Infertility duration, endometrial thickness and number of embryos transferred might affect the live birth rate after frozen embryo transfer among young women. This result could help inform clinical decisions and counseling to increase the live birth rate after frozen embryo transfer among young women (24). Therefore, although endometrial thickness has some impact on FET outcomes, its role in different age groups still requires further research.

This study has several limitations. First, its retrospective design and single-center setting may introduce selection bias and limit generalizability. Second, some confounding variables, such as embryo genomic stability and hormone levels, were not fully controlled, which could influence the results. Third, grouping embryos based on blastomere division provided useful insights but was somewhat subjective and did not fully assess long-term developmental potential. Additionally, the study lacked long-term follow-up data, leaving the extended impact of embryo thawing and culture on pregnancy outcomes unexamined. Lastly, compacted embryos were included in the cleavage-stage analysis without separate classification to focus on overall developmental potential and clinical outcomes. While the compaction stage could provide additional insights, this study aimed to evaluate the broader implications of extended culture. Future research should consider detailed classifications to explore this further.

In conclusion, this study indicates that D3 embryos with an increase in the number of blastomeres to more than four or entering the compaction stage after overnight culture have better pregnancy outcomes. Female age and endometrial thickness are important factors influencing clinical pregnancy rates. Optimizing culture conditions and ensuring optimal endometrial thickness may help improve the success rate of frozen-thawed embryo transfer.

## Data Availability

All relevant data is contained within the article: The original contributions presented in the study are included in the article, further inquiries can be directed to the corresponding authors.

## References

[1] Lawrenz B Coughlan C Melado L Fatemi HM. The ART of frozen embryo transfer: back to nature! Gynecol Endocrinol. (2020) 36:479–83. doi: 10.1080/09513590.2020.1740918 32188299

[2] von-Versen-Höynck F Conrad KP Baker VL. Which protocol for frozen-thawed embryo transfer is associated with the best outcomes for the mother and baby? Fertil Steril. (2021) 115:886–7. doi: 10.1016/j.fertnstert.2021.01.042 PMC8268856 33715870

[3] Bosch E De Vos M Humaidan P. The future of cryopreservation in assisted reproductive technologies. Front Endocrinol (Lausanne). (2020) 11:67. doi: 10.3389/fendo.2020.00067 32153506 PMC7044122

[4] Roque M Haahr T Geber S Esteves SC Humaidan P. Fresh versus elective frozen embryo transfer in IVF/ICSI cycles: a systematic review and meta-analysis of reproductive outcomes. Hum Reprod Update. (2019) 25:2–14. doi: 10.1093/humupd/dmy033 30388233

[5] Tocariu R Niculae LE Niculae AȘtefan Carp-Velișcu A Brătilă E. Fresh versus frozen embryo transfer in *in vitro* fertilization/intracytoplasmic sperm injection cycles: A systematic review and meta-analysis of neonatal outcomes. Medicina (Kaunas). (2024) 60:1373. doi: 10.3390/medicina60081373 39202656 PMC11356234

[6] Chang C-T Weng S-F Chuang H-Y Hsu I-L Hsu C-Y Tsai E-M. Embryo transfer impact: a comprehensive national cohort analysis comparing maternal and neonatal outcomes across varied embryo stages in fresh and frozen transfers. Front Endocrinol (Lausanne). (2024) 15:1400255. doi: 10.3389/fendo.2024.1400255 38933826 PMC11199782

[7] Guo YH Shen XX Liu Y Qi L Zhang XY Jin DC. Influencing factors analysis on live birth outcome of D3 cleavage stage frozen-thawed embryo after overnight culture and development of nomogram prediction model. Zhonghua Yi Xue Za Zhi. (2022) 102:877–83. doi: 10.3760/cma.j.cn112137-20211127-02658 35330582

[8] Wang J Ma L Mei J Li L Xu W Jiang W. Impacts of different culture times on pregnancy outcomes after thawing of cleavage stage embryos. BMC Pregnancy Childbirth. (2023) 23:824. doi: 10.1186/s12884-023-06139-7 38031033 PMC10685551

[9] Agha-Rahimi A Omidi M Akyash F Faramarzi A Farshchi FA. Does overnight culture of cleaved embryos improve pregnancy rate in vitrified-warmed embryo transfer programme? Malays J Med Sci. (2019) 26:52–8. doi: 10.21315/mjms2019.26.2.6 PMC6687221 31447608

[10] Ranji GG Shankar K Asokan Y Veerasigamani G Vittal RG Naaram NM. Impact of post-thaw incubation time of frozen embryos on clinical pregnancy rate. J Hum Reprod Sci. (2023) 16:64–9. doi: 10.4103/jhrs.jhrs_180_22 PMC10256942 37305777

[11] Ziebe S Bech B Petersen K Mikkelsen AL Gabrielsen A Andersen AN. Resumption of mitosis during post-thaw culture: a key parameter in selecting the right embryos for transfer. Hum Reprod. (1998) 13:178–81. doi: 10.1093/humrep/13.1.178 9512253

[12] Hwang JY Park JK Kim TH Eum JH Song H Kim JY. The impact of post-warming culture duration on clinical outcomes of vitrified-warmed single blastocyst transfer cycles. Clin Exp Reprod Med. (2020) 47:312–8. doi: 10.5653/cerm.2020.03832 PMC7711098 33181011

[13] Fang C Tang J Huang R Li L Zhang M Liang X. Comparison of amino acid metabolism in frozen-thawed and fresh early-stage human embryos. J Obstet Gynaecol Res. (2013) 39:1179–89. doi: 10.1111/jog.12035 23551919

[14] Li X Zeng Y He J Luo B Lu X Zhu L. The optimal frozen embryo transfer strategy for the recurrent implantation failure patient without blastocyst freezing: thawing day 3 embryos and culturing to day 5 blastocysts. Zygote. (2023) 31:596604. doi: 10.1017/S0967199423000503 37969109

[15] Agarwal A Maldonado Rosas I Anagnostopoulou C Cannarella R Boitrelle F Munoz LV. Oxidative stress and assisted reproduction: A comprehensive review of its pathophysiological role and strategies for optimizing embryo culture environment. Antioxidants (Basel). (2022) 11:477. doi: 10.3390/antiox11030477 35326126 PMC8944628

[16] Zhu H Xu W Jin X Xue Y Tong X Zhang S. Association of the duration of post-thaw culture with clinical outcome after vitrified-warmed day 3 embryo transfer in 10,464 cycles: A retrospective cohort study. Med (Baltimore). (2020) 99:e21660. doi: 10.1097/MD.0000000000021660 PMC7437799 32872029

[17] Li Y Cai X Li N Zhang L Ma B. The effects of different post-thawed culture periods on clinical outcomes in frozen embryo transfer cycle. Reprod Sci. (2022) 29:936–43. doi: 10.1007/s43032-021-00760-7 34642911

[18] Van der Elst J Van den Abbeel E Vitrier S Camus M Devroey P Van Steirteghem AC. Selective transfer of cryopreserved human embryos with further cleavage after thawing increases delivery and implantation rates. Hum Reprod. (1997) 12:1513–21. doi: 10.1093/humrep/12.7.1513 9262288

[19] Nahshon C Dirnfeld M Koifman M Blais I Lahav-Baratz S. Comparison of day 2 and overnight day 3 frozen embryo transfers: A prospective randomized controlled trial. Reprod Biol. (2021) 21:100565. doi: 10.1016/j.repbio.2021.100565 34600346

[20] Holschbach V Kordes H Dietrich JE Bruckner T Strowitzki T Germeyer A. Patient- and cycle-specific factors affecting the outcome of frozen-thawed embryo transfers. Arch Gynecol Obstet. (2023) 307:2001–10. doi: 10.1007/s00404-023-07019-3 PMC10147814 37061986

[21] Glujovsky D Quinteiro Retamar AM Alvarez Sedo CR Ciapponi A Cornelisse S Blake D. Cleavage-stage versus blastocyst-stage embryo transfer in assisted reproductive technology. Cochrane Database Syst Rev. (2022) 5:CD002118. doi: 10.1002/14651858.CD002118.pub6 35588094 PMC9119424

[22] Laverge H van der Elst J De Sutter P Verschraegen-Spae MR De Paepe A Dhont M. Fluorescent *in-situ* hybridization on human embryos showing cleavage arrest after freezing and thawing. Hum Reprod. (1998) 13:425–9. doi: 10.1093/humrep/13.2.425 9557851

[23] Tan C Wang X Luo L Zhang J Zou P Wei W. The feasibility of choosing D4 embryo transfer-analysis of nanomaterials affecting the outcome of frozen-thaw embryo transfer. Evid Based Complement Alternat Med. (2022) 2022:1364865. doi: 10.1155/2022/1364865 36262164 PMC9576394

[24] Pan Y Hao G Wang Q Liu H Wang Z Jiang Q. Major factors affecting the live birth rate after frozen embryo transfer among young women. Front Med (Lausanne). (2020) 7:94. doi: 10.3389/fmed.2020.00094 32266278 PMC7105776

